# Factors influencing cervical cancer screening practice among female health workers in Nigeria: A systematic review

**DOI:** 10.1002/cnr2.1514

**Published:** 2021-07-27

**Authors:** Elvis Anyaehiechukwu Okolie, Debra Barker, Lawrence Achilles Nnyanzi, Seun Anjorin, David Aluga, Blessing Ifeoma Nwadike

**Affiliations:** ^1^ School of Health and Life Sciences Teesside University Middlesbrough UK; ^2^ Division of Health Sciences Warwick Medical School, University of Warwick Coventry UK; ^3^ Department of Microbiology University of Ibadan Ibadan Nigeria

**Keywords:** cancer screening, cervical cancer, female health workers, Nigeria

## Abstract

**Background:**

Cervical cancer is the most prevalent gynaecologic cancer in Nigeria. Despite being largely preventable through screening, cervical cancer is the second leading cause of cancer morbidity and mortality in Nigeria. To reduce the burden of cervical cancer in Nigeria, female health workers (FHWs) are expected to play an influential role in leading screening uptake and promoting access to cervical cancer education and screening.

**Aim:**

The aim of this systematic review is to assess the factors influencing cervical cancer screening (CCS) practice among FHWs in Nigeria.

**Methods:**

We conducted a systematic literature search across six (6) electronic databases namely MEDLINE, Embase, Scopus, African Index Medicus, CINAHL, and Web of Science between May 2020 and October 2020. Reference list and grey literature search were conducted to complement database search. Four reviewers screened 3171 citations against the inclusion criteria and critically appraised the quality of eligible studies. Narrative synthesis was used in summarising data from included studies.

**Results:**

Overall, 15 studies met the inclusion criteria and were all quantitative cross‐sectional studies. Included studies sampled a total of 3392 FHWs in Nigeria. FHWs had a high level of knowledge and positive attitude towards CCS. However, CCS uptake was poor. Predominant barriers to CCS uptake were the cost of screening, fear of positive results, lack of test awareness, reluctance to screen, low‐risk perception, and lack of time. In contrast, being married, increasing age, awareness of screening methods, and physician recommendation were the most documented facilitators.

**Conclusion:**

This study revealed that a complex interplay of socioeconomic, structural, and individual factors influences CCS among FHWs in Nigeria. Therefore, implementing holistic interventions targeting both health system factors such as cost of screening and infrastructure and individual factors such as low‐risk perception and fear of positive result affecting FHWs in Nigeria is critical to reducing the burden of cervical cancer.

## 1 INTRODUCTION

Cervical cancer represents a significant threat to reducing global health inequalities and achieving sustainable development goals. This disease is the commonest gynaecological cancer affecting women especially in low and middle‐income countries (LMICs).^1^ Despite being largely preventable, an estimated 570 000 cervical cancer cases and 311 000 deaths from the disease occurred in 2018.^2^ It is frightening to note that over 85% of cervical cancer incidence and mortality occur in LMICs including Nigeria where organised population cervical cancer screening (CCS) programmes are inadequate and treatment options limited.^3^, ^4^ In contrast, high‐income countries have witnessed almost 70% decrease in cervical cancer burden over the last 50 years upon the introduction of organised CCS programmes.^5^ Such disparities between countries demonstrate stark inequalities in healthcare resources and enduring socioeconomic barriers especially in LMICs.^6^, ^7^

In Nigeria, cervical cancer is the second principal cause of cancer morbidity and mortality with an estimated incidence of 14 943 cases and 10 403 deaths in 2018.^2^ In the absence of improvements to current cervical cancer prevention strategies in Nigeria, an estimated 51 million women aged 15 and above will be at risk of developing cervical cancer.^8^ The enormity of the impact of cervical cancer observed in terms of man‐hour loss and medical costs results in about $3.3 million/disability adjusted life years lost annually.^9^ The burden placed on women by this disease contributes to the perpetuation of poverty and disruption of the socio‐economic fabric of both families and communities.^7^, ^9^

Fortunately, we are witnessing a shift from the fatalistic acceptance of cervical cancer to cautious optimism for its elimination due to growing knowledge of the natural history of disease and advancements in prevention.^10^, ^11^ The natural history of cervical cancer allows for multiple interventions – primary, secondary, and tertiary.^12^ First, the well‐established evidence that persistent infection with high‐risk Human Papillomavirus (HPV) subtypes is the principal causal factor in 99.7% of all cervical cancer cases^13^ stimulated vaccine development.^14^ Despite promising results from HPV vaccination, glaring inequalities in vaccine access and failure of vaccines to protect against all cancer‐inducing HPV strains makes screening the best‐buy in the continuum of interventions against cervical cancer.^15^, ^16^, ^17^ Three major CCS methods are the Papanicolaou smear test, HPV‐based testing, and visual inspection with acetic acid (VIA).^18^, ^19^, ^20^

Regrettably, LMICs including Nigeria are lagging in the implementation of organised CCS using any of the methods due to various challenges; absence of national CCS policies and guideline, paucity of resources, weak political commitment, and deficient health systems.^21^, ^22^, ^23^, ^24^ Consequently, available CCS services in Nigeria are mostly opportunistic, inequitably distributed, and reach a small proportion of eligible women.^25^ It becomes worrisome knowing that only 8.7% of all eligible women have been reached with opportunistic screening in Nigeria.^26^ Such poor screening rate has been linked to a spectrum of factors; weak health system, poor awareness, low‐risk perception, sociocultural barriers, fear of positive result, poverty, and acceptability of available screening options.^27^, ^28^, ^29^, ^30^ Despite the emphasis on taking advantage of women's contact with the health system to provide CCS services, evidence indicates that such opportunities have been missed.^28^, ^31^

Given the poor screening status of women in Nigeria, calls for addressing these missed opportunities for CCS have been made.^1^, ^25^, ^31^ At the core of efforts to improve screening uptake lie female health workers (FHWs). Evidence demonstrates that health personnel recommendation is a key driver of CCS uptake especially in situations where motivation may be inadequate.^32^, ^33^ For instance, Okunowo and Smith‐Okonu^33^ found that 53% of women who received CCS in a secondary facility in Lagos reported recommendation by doctor/nurse as a key motivating factor. Undoubtedly, FHWs as role models in healthcare are expected to facilitate a supportive environment that encourages women to utilise screening opportunities.^34^ Equally important is that the profession of FHWs does not preclude them from the risk associated with cervical cancer.

Therefore, understanding determinants such as cervical cancer‐related knowledge, attitudes, and screening practices among FHWs could improve overall screening uptake by informing policy initiatives and intervention design.^35^ This study is the first systematic review aimed at synthesising and generating robust evidence on the factors influencing CCS uptake among FHWs in Nigeria. Additionally, this systematic review will highlight CCS related knowledge, attitudes, and practices which are valuable in improving screening uptake for the general population.

## 2 METHODOLOGY

The method adopted for this study was informed by the guidelines contained in the Centre for Reviews and Dissemination (CRD) guidance for undertaking reviews in healthcare^36^ and The Cochrane Handbook for Systematic Reviews Version 6.1.^37^ This study is reported in accordance with the preferred reporting items for systematic reviews and meta‐analyses (PRISMA) guidelines.^38^ The protocol (https://www.crd.york.ac.uk/prospero/display_record.php?ID=CRD42020186750) for this study is registered with The International Prospective Register of Systematic Reviews (PROSPERO).

### 2.1 Search strategy and data sources

The systematic search for primary studies relevant to the review question ‘what are the factors influencing CCS uptake among FHWs in Nigeria’ included keywords and related terms derived from scoping search and entry terms of Medical Subject Headings (MeSH). These terms were combined with Boolean operators to ensure balanced sensitivity and precision during database search. These terms include; Female health* workers OR Health personnel* OR Nurs* AND Cervical screening OR Early detection of cancer OR Pap* smear OR HPV testing AND Awareness OR Attitudes OR Practices OR Determin* OR Access OR Facilitators OR Barriers OR Socioeconomic AND Nigeria* OR Sub Sahara* Africa OR Low and middle‐income countr*. Six (6) electronic databases namely MEDLINE, Embase, CINAHL, Scopus, African Index Medicus, and Web of Science were searched between May to June 2020 and a repeat search conducted in October 2020. We utilised unique syntax and symbols (truncations or wildcards) to maintain consistency in search across selected databases. To ensure the rigour of our search in obtaining relevant primary studies, we identified key papers that met set inclusion criteria before conducting database search. Upon obtaining the search results, these key papers were identified showing that our search was robust.^39^

Furthermore, we carried out a supplementary search for grey literature and studies not indexed in selected databases using Google and Google Scholar. The first 15 pages of results were retained and examined for relevant primary studies. Reference list search of all included studies was conducted to identify related articles. No time or language restrictions were applied in the course of systematic search to allow for rigour. Detailed search strategy and outcomes from selected databases are attached as appendices (Tables A1, A2, A3, A4, A5, A6, A7).

### 2.2 Eligibility criteria

The inclusion and exclusion criteria for this systematic review were informed by the PIOS‐based review question. The eligibility criteria for this study are delineated in Table 1. Included studies in this systematic review met all the inclusion criteria and none of the exclusion criteria. Specifically, this systematic review focused on quantitative studies reporting outcomes of interest such as knowledge, attitude, practices, and factors influencing CCS among FHWs in Nigeria.

**TABLE 1:** Eligibility criteria for the systematic review

S/n	Parameters	Inclusion criteria	Exclusion criteria
1	Study population (P)	Studies focusing on FHWs irrespective of cadre	Studies focusing on general women population or non‐FHWs
2	Intervention (I)	Studies focusing on CCS	Studies focusing on primary prevention (vaccination) or tertiary prevention approaches to cervical cancer
3	Study focus (O)	Studies reporting factors influencing CCS uptake	Studies not reporting barriers or facilitators of CCS uptake
4	Study location (S)	Studies conducted in Nigeria	Studies conducted outside of Nigeria
5	Study design	Observational studies with either quantitative or mixed‐method study design with distinctive quantitative reporting of the outcomes of interest	Observational studies with qualitative design or mixed‐method study design without distinctive quantitative reporting of the outcomes of interest
6	Access to full text	Studies that are accessible and available in full text	Studies with insufficient information on methodology and outcomes of interest due to full‐text restriction.

Abbreviations: CCS, cervical cancer screening; FHWs, female health workers.

### 2.3 Study selection

**FIGURE 1:**
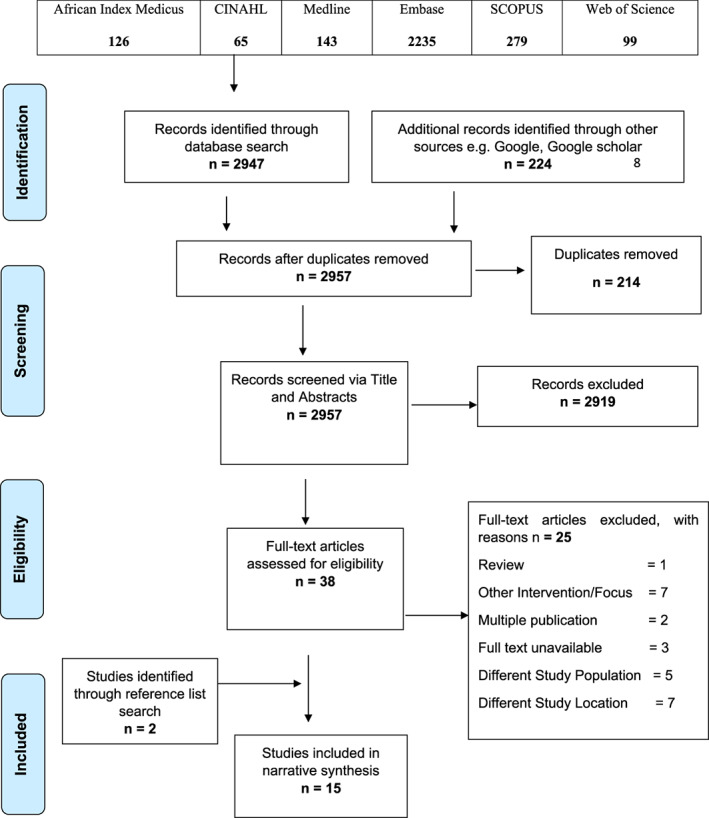
Preferred reporting items for systematic reviews and meta‐analyses flow diagram showing the study selection process

All studies obtained from database and grey literature search were saved in Zotero Library version 5.0.84 for storage, duplicate removal, and study selection based on predetermined eligibility criteria. A total of 3171 citations were retrieved from the systematic search. After duplicate removal, we utilised the two‐stage recommendation of the CRD in study selection: (a) Initial screening of titles and abstracts against the eligibility criteria to identify relevant papers and (b) Screening of full‐text papers identified as potentially relevant from the first stage.^36^ Four independent reviewers (O.E., A.S., A.D., N.B.) were employed across these stages (two for each stage) to allow for reliability and avoid reviewer fatigue.^40^ Studies that did not meet the inclusion criteria were excluded and reasons for such exclusion stated (Appendix 2). Efforts were made to obtain relevant papers in this study through the Teesside University Library and contacting authors; three papers were not available after these efforts. Discrepancies between reviewers was discussed and resolved through a consensus.^41^ The PRISMA four‐phase flow diagram was used in reporting study selection processes (Figure 1).

### 2.4 Study quality appraisal

The quality of all the primary studies included in this systematic review was critically appraised independently by two reviewers (O.E. and B.D.) using the AXIS tool checklist for Cross‐sectional studies (Appendix 3).^42^ Any disagreement in quality assessment was resolved through discussion. Specifically, the AXIS tool for cross‐sectional studies is a 20‐point questionnaire directed at the quality of study design, reporting, and risk of bias.^42^ The key focus of the AXIS tool include (a) study design; (b) sample size justification; (c) target population; (d) sampling frame; (e) sample selection; (f) measurement validity and reliability; (g) overall methods. Quality appraisal outcomes for included studies were categorised as low, moderate, and high quality. These categories were informed by the level of description of study design and clarity in reporting study components including the risk of bias.

### 2.5 Data extraction

Data from included studies was extracted using a predetermined Microsoft Excel data extraction table adapted from the Cochrane Data collection form to suit the objectives of our review (Appendix 4). Data extracted from studies included: (a) bibliographic information; (b) study objective; (c) study design (methodology and sample size); (d) participant characteristics (socio‐demographic variables); (e) results or key findings; (f) conclusion including the recommendation for research or practice.^43^ Data extraction was carried out by one reviewer (O.E.) and subsequently assessed by a second reviewer (A.S.) to ensure quality. Any observed discrepancies were addressed through discussion.

### 2.6 Data analysis and synthesis

A critical evaluation of included studies revealed their unsuitability for meta‐analysis due to statistical heterogeneity arising from variation in measurements.^36^, ^37^ Consequently, a narrative synthesis of quantitative data was adopted in synthesising results from included studies.^44^ In applying the narrative synthesis approach, the study characteristics and findings from included studies were summarised and contextually described to answer the review question. The results are presented as textual narratives in combination with tables highlighting relevant outcomes. The primary outcome measurements to be analysed in this systematic review are (a) Knowledge of CCS: This refers to an understanding of the benefits of CCS. (b) Attitude towards screening: This includes the willingness to engage in screening or intention to recommend screening to other women. (c) CCS practices: This refers to the utilisation of CCS services. (d) Barriers to CCS: This refers to reasons for not utilising screening services e. Facilitators of CCS: This refers to factors enabling the use of screening services.

## 3 RESULT

A systematic search of six electronic databases (*n* = 2947) and grey literature sources such as Google and Google scholar (*n* = 224) yielded a total of 3171 citations (Figure 1). At the end of duplicate removal using Zotero, 2957 unique citations were included for screening via titles and abstract. After initial title and abstract screening to identify potentially relevant papers that answer the review question and fits the inclusion criteria, 2919 citations that did not fit the inclusion criteria or answer the review question were removed and 38 citations were retained for full‐text screening to determine their eligibility for final inclusion. Upon the application of inclusion criteria and full‐text review by two independent reviewers (O.E. and A.S.), 13 studies were included for the review. Furthermore, reference list search identified an additional two papers bringing the total number of included studies to 15.^45^, ^46^, ^47^, ^48^, ^49^, ^50^, ^51^, ^52^, ^53^, ^54^, ^55^, ^56^, ^57^, ^58^, ^59^ Reasons for exclusion of papers were: different study location (*n* = 7), different study population (*n* = 5), multiple publication (*n* = 2), focus on other interventions (*n* = 7), full text unavailable (*n* = 3), and review (*n* = 1).

### 3.1 Study characteristics

Summary characteristics of all included primary studies are displayed in Table 2. All included studies (*n* = 15) were quantitative cross‐sectional studies. Similarly, questionnaires were the key data collection for all included studies. The 15 studies included in this systematic review sampled a total of 3392 FHWs; study sample sizes ranged from 40 to 503.^52^, ^56^ In determining study sample sizes, only 10 studies documented the justification for selecting a particular sample size.^46^, ^48^, ^49^, ^50^, ^52^, ^53^, ^54^, ^55^, ^57^, ^58^ Majority of the studies (*n* = 8) included different cadres of FHWs,^45^, ^46^, ^48^, ^53^, ^54^, ^56^, ^57^, ^58^ two studies involved only medical doctors,^49^, ^55^ and five focused solely on nurses.^47^, ^50^, ^51^, ^52^, ^59^ The age of participants in selected studies was measured in a range between 20 and 60 years.

**TABLE 2:** Description of included studies

S/n	Author, year	Title, location	Sample size	Study participants	Study quality
1	Ayinde and Omigbodun (2003)	Knowledge, attitude, and practices related to prevention of cancer of the cervix among female health workers in Ibadan (Ibadan)	205	Doctors, nurses, and hospital maids.	Moderate
2	Aboyeji et al. (2004)	Knowledge, Attitude and Practice of Cervical Smear as a Screening Procedure for Cervical Cancer in Ilorin, Nigeria (Ilorin)	483	Doctors, nurses, medical lab scientists, and pharmacists.	Moderate
3	Udigwe (2006)	Knowledge, attitude, and practice of cervical cancer screening (pap smear) among female nurses in Nnewi, South Eastern Nigeria (Nnewi)	140	Nurses	Moderate
4	Gharoro and Ikeanyi (2006)	An appraisal of the level of awareness and utilisation of the Pap smear as a cervical cancer screening test among female health workers in a tertiary health institution (Benin)	194	Doctors, nurses, lab technicians, pharmacists, radiographers, and hospital maids	Low
5	Dim et al. (2009)	Improved awareness of Pap smear may not affect its use in Nigeria: a case study of female medical practitioners in Enugu, South‐Eastern Nigeria (Enugu)	80	Doctors	Moderate
6	Awodele et al. (2011)	A Study on Cervical Cancer Screening Among Nurses in Lagos University Teaching Hospital, Lagos, Nigeria (Lagos)	200	Nurses	Moderate
7	Unang et al. (2011)	Awareness and Practice of Cervical Smear as A Screening Procedure for Cervical Cancer among Female Nurses in A Tertiary Hospital in South–South Nigeria (Uyo)	176	Nurses	High
8	Arulogun and Maxwell (2012)	Perception and utilisation of cervical cancer screening services among female nurses in University College Hospital, Ibadan, Nigeria (Ibadan)	503	Nurses	Moderate
9	Oche et al. (2013)	Cancer of the cervix and cervical cancer screening: Current knowledge, attitude, and practices of female health workers in Sokoto, Nigeria (Sokoto)	240	Doctors, nurses, lab. scientists, and pharmacists	High
10	Takai et al. (2015)	Awareness and utilisation of Papanicolaou smear among health care workers in Maiduguri, Nigeria (Maiduguri)	150	Doctors, nurses, pharmacists, medical lab. scientists, and others	Moderate
11	Jagun et al. (2016)	Uptake of Cervical Cancer Screening Services Among Female Medical Practitioners in Ogun State, South‐West Nigeria (Ogun)	85	Doctors	Moderate
12	Daniyan et al. (2019)	Assessment of Knowledge, Attitudes and Practice of Cervical Cancer Screening Among Female Health Workers in a Tertiary Health Facility in South‐East Nigeria (Abakiliki)	40	FHWs	Low
13	Omonua et al. (2019)	A Study on the Awareness and Utilisation of Pap Smear Among Female Health Workers in a Tertiary Hospital in Nigeria (Abuja)	223	Doctors, nurses, pharmacists	Moderate
14	Awoyesuku et al. (2019)	Knowledge, Uptake and Barriers to Pap Smear Test among Female Workers in the Rivers State University Teaching Hospital, Nigeria (Rivers)	265	Doctors, nurses, medical lab. Scientists, pharmacists, and others	Moderate
15	Ifemelumma et al. (2019)	Cervical Cancer Screening: Assessment of Perception and Utilisation of Services among Health Workers in Low Resource Setting (Abakaliki)	408	Nurses	High

Included primary studies were published between 2003 and 2019; with the highest number of the studies (*n* = 4) being published in 2019. While time limit was not applied, systematic search for relevant studies was delimited to Nigeria. Analysis of study location for included studies demonstrated country‐wide coverage as each of the six geopolitical zones had at least one study; North Central (*n* = 2; Abuja and Ilorin), North East (*n* = 1; Maiduguri), Northwest (*n* = 1; Sokoto), South East (*n* = 4; Awka, Enugu, and two studies in Abakaliki), South‐South (*n* = 3; Benin, Uyo, and Rivers), and South‐West (*n* = 4; Lagos, Ogun, and two studies in Ibadan). Majority of included studies were conducted in tertiary health institutions (*n* = 10), three were multi‐centred studies involving a mix of tertiary and secondary health facilities, and two focused on professional associations (Medical Women Association).

### 3.2 Quality assessment

Majority of included studies (*n* = 10) were moderate in terms of study quality, two were categorised as having low quality, and three studies were regarded as having high quality (Table 2). Predominant methodological factors responsible for weakness in appraised studies include non‐justification of sample size, poor documentation of the reliability and validity of data collection tools, and non‐disclosure of risk of bias in study outcomes. For instance, the justification of study sample size is recognised as good practice and is crucial in identifying the existence and magnitude of an effect.^60^

Furthermore, only two studies^53^, ^54^ out of the 15 included discussed the limitations of their study. About half of the studies (*n* = 7) disclosed either funding sources or conflict of interest that may influence author interpretation of findings.

### 3.3 Awareness and knowledge of CCS among FHWs

Knowledge and/or awareness of CCS was a key outcome measurement across selected studies. Awareness of CCS focused on general information about its existence; have FHWs heard of CCS? While knowledge of CCS focused on assessment of specific information about the benefits of CCS among FHWs. Five studies reported only awareness of CCS,^47^, ^48^, ^49^, ^54^, ^55^ seven studies reported both awareness and knowledge of CCS,^50^, ^51^, ^52^, ^53^, ^57^, ^58^, ^59^ and three studies reported only knowledge.^45^, ^46^, ^56^ Eight studies reported knowledge outcomes as proportions (*n* = 8) while two studies^45^, ^56^ measured knowledge using a Likert scale. An understanding of the use and benefits of CCS was a measurement criterion for screening knowledge.

Overall, awareness and knowledge of CCS use were high across selected studies (Table 3). Awareness among FHWs ranged from 61^55^ to 100%.^49^, ^59^ Similarly, knowledge levels ranged from 54.5^52^ to 90.5%.^53^ For studies that used a Likert scale, mean knowledge scores ranged from 3.32 to 6.47 out of 8 in Ayinde and Omigbodun^45^ and from 4.55 to 4.68 out of five in Daniyan et al.^56^ Beyond the knowledge of the use screening, selected studies assessed other areas of screening knowledge. Particularly, knowledge of screening interval, target population, screening results, and screening methods was investigated.

**TABLE 3:** Summary of CCS outcome measurements

S/n	Author, year	Knowledge/awareness of CCS	Attitudes towards CCS	CCS practices
1	Ayinde and Omigbodun, (2003)	Mean knowledge scores: Doctors = 6.47/8, Nurses = 4.72/8, Hospital maids = 3.32/8	Willingness to test = Yes (53.9%)	Ever had a pap smear = Yes (6.8%)
2	Aboyeji et al. (2004)	Knowledge = Yes (69.8%)	Willingness to test = Yes (19.6%)	Ever had a pap smear = Yes (3%)
3	Udigwe, (2006)	Awareness = Yes (87.1%)	NA	Ever had a pap smear = Yes (5.7%)
4	Gharoro and Ikeanyi, (2006)	Awareness = Yes (64.7%)	Willingness to test = Yes (64.7%)	Ever had a pap smear = Yes (14.1%)
5	Dim et al. (2009)	Awareness = Yes 100%	NA	Ever had a pap smear = Yes (18%)
6	Awodele et al. (2011)	Awareness = Yes (91%) Knowledge = Yes (60%)	Perception of screening importance = Yes (89%) Routine recommendation = Yes (34%)	Ever had a pap smear = Yes (21.5%)
7	Unang et al. (2011)	Awareness = Yes (94.3%) Knowledge = Yes (79.5%)	NA	Ever had a pap smear = Yes (7.4%)
8	Arulogun and Maxwell (2012)	Awareness = Yes (80.9%) Knowledge = Yes (54.5%)	Willingness to test = Yes (81%)	Ever had a pap smear = Yes (34.5%)
9	Oche et al. (2013)	Awareness = Yes (98.6%) Knowledge = Yes (90.5%)	Willingness to test = Yes (77.7%) Intention to recommend screening = Yes (81.9%)	Ever had a pap smear = Yes (10%)
10	Takai et al. (2015)	Awareness = Yes (94%)	Willingness to test = Yes (70.6%)	Ever had a pap smear = Yes (23.3%)
11	Jagun et al. (2016)	Awareness = Yes (61%)	NA	Ever had a pap smear = Yes (39.8%)
12	Daniyan et al. (2019)	Mean knowledge scores 4.55–4.68/5.	Perception of screening importance = 4.39–4.81/5.	Ever had a pap smear = Yes (54.1%)
13	Omonua et al. (2019)	Awareness = Yes (97.5%) Knowledge = Yes (58%)	NA	Ever had a pap smear = Yes (23.5%)
14	Awoyesuku et al. (2019)	Awareness = Yes (89.4%) Knowledge = Yes (78.5%)	NA	Ever had a pap smear = Yes (16.9%)
15	Ifemelumma et al. (2019)	Awareness = 100%, Knowledge = Yes (89.2%)	Routine recommendation = Yes (43.3%)	Ever had a pap smear = Yes (20.6%)

Abbreviations: CCS, cervical cancer screening; NA, not assessed.

FHWs knowledge on recommended CCS interval and target population eligible for screening was found to be poor.^46^, ^50^, ^57^, ^59^ Similarly, an understanding of screening results among FHWs was found to be inadequate.^50^, ^57^ For studies that assessed knowledge of screening methods, pap smears were reported as the most popular screening method. Among studies that stratified CCS knowledge by cadre of FHWs, knowledge was observed to be ‘profession‐dependent’ as doctors and nurses were more knowledgeable compared to others.^45^, ^48^

Furthermore, 60% of the studies (*n* = 9) reported sources of CCS information for FHWs. Prevalent sources of information reported by FHWs across selected studies include media, school lectures/medical training, health professionals/colleagues, seminar, and friends.

### 3.4 Attitude of FHWs towards CCS

Out of the 15 included studies for this systematic review, 60% (*n* = 9) assessed the attitude of FHWs towards CCS (Table 3). Core information elicited by studies investigating attitude of FHWs towards CCS includes the willingness to test (*n* = 6),^45^, ^46^, ^48^, ^52^, ^53^, ^54^ perception of screening importance (*n* = 2),^50^, ^56^ and intention to recommend/routine recommendation of CCS (*n* = 3).^50^, ^53^, ^59^ Overall, the majority of the studies reported positive attitude towards CCS among FHWs.

In terms of FHWs willingness to screen, the majority (*n* = 5) of the studies reported high willingness to have a CCS test ranging from 53.9% in Ayinde and Omigbodun^45^ to 81% in Arulogun and Maxwell.^52^ Conversely, Aboyeji et al^46^ documented negative attitude among FHWs as 77.4% were unwilling to participate in screening due to low‐risk perception. Overall, perception towards the importance of CCS was good as 89% of FHWs in Awodele et al^50^ opined that it is advisable to screen. Similarly, Daniyan et al^56^ reported an attitude range of 4.39–4.81 on a scale of five indicating that the majority of sampled FHWs perceived CCS as beneficial.

Furthermore, a wide gap was observed between the intention to recommend CCS and actual recommendation practices among FHWs. While reported intention to recommend screening to others was as high as 81.9%,^53^ low practice of routine screening recommendation ranging from 34 to 43.3% was documented.^50^, ^59^

### 3.5 CCS practices among FHWs

All 15 studies for this systematic review assessed CCS practices among FHWs (Table 3). The previous history of CCS uptake among FHWs was a key practice indicator across selected studies. Overall, a trend of poor utilisation of CCS was observed among FHWs. The proportion of FHWs that have previously screened ranged from 3% in Aboyeji et al^46^ to 54.1% in Daniyan et al.^56^ In contrast, the proportion of FHWs without any history of CCS was high, ranging from 45.9 to 97%.

Again, among studies that stratified screening practices by cadre of FHWs, a significant association was observed between CCS uptake and the cadre of FHWs.^46^, ^48^ In Aboyeji et al,^46^ screening uptake was significantly different between doctors (6.5%) and other FHWs such as medical laboratory scientists (4.3%) and nurses (1.7%). Gharoro and Ikeanyi^48^ documented higher screening rates (73.1%) among nurses compared to hospital maids (0%) who had little or no medical training.

### 3.6 CCS related barriers and facilitators

Fourteen studies documented barriers to CCS uptake among FHWs. A total of 20 barriers were identified as key reasons for non‐utilisation of cervical cancer services among FHWs in Nigeria (Table 4). These barriers could be broadly categorised into health system and individual‐level barriers. Individual‐level barriers refer to those factors impeding the uptake of CCS at the level of the individual FHW. Prevalent individual‐level barriers reported across primary studies include fear of positive result, low‐risk perception, lack of test awareness/ignorance, and lack of time/being busy.

**TABLE 4:** Barriers to CCS uptake

S/n	Reported barriers	No. of studies	Author(s)
1	Cost of screening	*n* = 9	Ayinde and Omigbodun (2003), Aboyeji et al. (2004); Udigwe (2006); Awodele et al. (2011); Unang et al. (2011); Arulogun and Maxwell (2012); Takai et al. (2015); Jagun et al. (2016); Awoyesuku et al. (2019)
2	Fear of positive result	*n* = 9	Aboyeji et al. (2004), Udigwe (2006), Dim et al. (2009), Unang et al. (2011), Arulogun and Maxwell (2012), Oche et al. (2013), Jagun et al. (2016), Ifemelumma et al. (2019), Awoyesuku et al. (2019),
3	Lack of test awareness/ignorance	*n* = 8	Ayinde and Omigbodun (2003); Gharoro and Ikeanyi (2006), Udigwe (2006), Jagun et al. (2016), Awodele et al. (2011), Arulogun and Maxwell, (2012), Oche et al. (2013), Ifemelumma et al. (2019)
4	Low risk perception	*n* = 8	Ayinde and Omigbodun (2003), Aboyeji et al. (2004), Gharoro and Ikeanyi (2006), Udigwe (2006), Unang et al. (2011), Oche et al. (2013), Omonua et al. (2019).
5	Reluctance/no reason	*n* = 7	Ayinde and Omigbodun (2003), Aboyeji et al. (2004), Udigwe (2006), Dim et al. (2009), Awodele et al. (2011), Unang et al. (2011), Omonua et al. (2019)
6	Lack of time/being busy	*n* = 6	Dim et al. (2009), Awodele et al. (2011), Arulogun and Maxwell, 2012; Ifemelumma et al. (2019), Omonua et al. (2019), Awoyesuku et al. (2019).
7	Poor knowledge of testing facilities	*n* = 5	Awodele et al. (2011), Unang et al. (2011), Arulogun and Maxwell (2012), Takai et al. (2015), Ifemelumma et al. (2019).
8	Neglect of screening	*n* = 5	Awodele et al. (2011), Unang et al. (2011), Ifemelumma et al. (2019) Omonua et al. (2019), Awoyesuku et al. (2019)
9	Sexually inactive	*n* = 4	Ayinde and Omigbodun, 2003; Arulogun and Maxwell, 2012; Ifemelumma et al. (2019), Awoyesuku et al. (2019).
10	Lack of screening services	*n* = 3	Gharoro and Ikeanyi (2006), Jagun et al. (2016), Awoyesuku et al. (2019).
11	Religious/cultural beliefs	*n* = 2	Aboyeji et al. (2004), Gharoro and Ikeanyi (2006).
12	Laziness	*n* = 2	Dim et al. (2009), Jagun et al. (2016).
13	Cumbersome procedure	*n* = 2	Arulogun and Maxwell (2012), Ifemelumma et al. (2019)
14	Lack of money	*n* = 1	Awodele et al. (2011)
15	Husband disapproval	*n* = 1	Aboyeji et al. (2004)
16	Preservation of virginity	*n* = 1	Dim et al. (2009)
17	Being young	*n* = 1	Unang et al. (2011)
18	Lack of recommendation	*n* = 1	Takai et al. (2015)
19	Gender of screening provider	*n* = 1	Oche et al. (2013).
20	Fear of pain	*n* = 1	Oche et al. (2013)

Abbreviation: CCS, cervical cancer screening.

In contrast, barriers at the health system level refer to health system or service delivery factors that result in the exclusion of FHWs who may want to screen. Core institutional barriers reported across selected studies include the cost of screening, cumbersome nature of the procedure, lack of CCS recommendation, and gender of screening provider.

Furthermore, 53% of included studies (*n* = 8) reported facilitators of CCS among FHWs. Ten facilitators were identified (Table 5) as reasons for screening among FHWs who had previously screened. Major reasons for screening include being married, increasing age of the FHW, physician recommendation, and being ill.

**TABLE 5:** Facilitators of CCS uptake

S/n	Facilitators	No. of studies	Author(s)
1	Being married/marital status	*n* = 4	Aboyeji et al. (2004), Oche et al. (2013), Ifemelumma et al. (2019), Awoyesuku et al. (2019).
2	Increasing age	*n* = 4	Dim et al. (2009), Awodele et al. (2011), Oche et al. (2013), Ifemelumma et al. (2019).
3	Awareness of screening methods	*n* = 3	Udigwe, (2006), Awodele et al. (2011), Jagun et al. (2016).
4	Being Ill	*n* = 2	Dim et al. (2009), Jagun et al. (2016)
5	Physician recommendation	*n* = 2	Oche et al. (2013), Jagun et al. (2016)
6	Membership of clinical department	*n* = 1	Aboyeji et al. (2004)
7	Cadre of FHWs	*n* = 1	Arulogun and Maxwell (2012)
8	Higher educational attainment	*n* = 1	Takai et al. (2015)
9	Availability of screening services	*n* = 1	Jagun et al. (2016)
10	Parity	*n* = 1	Ifemelumma et al. (2019)

Abbreviation: CCS, cervical cancer screening.

## 4 DISCUSSION

This systematic review investigated factors influencing CCS uptake among FHWs in Nigeria. This study observed a high level of awareness and knowledge of the use of cervical screening among FHWs. While good knowledge of CCS use is fundamental to cervical cancer prevention, it is not surprising that a significant proportion of FHWs understood the need for screening.^50^, ^53^ By their profession, health workers are trained to respond to varying health challenges and are expected to be knowledgeable about cervical cancer‐related issues.^56^ Similarly, pap smear was the most popular screening method among FHWs.^52^, ^59^ For instance, Ifemelumma et al^59^ reported that 89.2% of FHWs knew pap smear compared to 41.2 and 25.5% with knowledge of VIA and HPV‐based testing, respectively. The popularity of pap smear over other methods may emanate from its longstanding use as the traditional screening method and/or gaps in the promotion of other screening methods.

While the benefits of screening were well understood by FHWs, this study revealed inadequate knowledge of screening interval, recommended target population, and interpretation of screening results among FHWs.^46^, ^50^, ^57^, ^59^ Such inadequacy in comprehensive screening knowledge highlights existing knowledge and competency gaps and raises concern on several factors influencing cervical cancer information available to FHWs; source, thoroughness, and coherency of information. Media, medical literature, health professionals, and school represented the major sources of cervical screening information for FHWs. Medical literature and school as an information source suggest the provision of fundamental cervical cancer education during the medical training of FHWs.^46^, ^58^ Similarly, FHWs reporting health professionals or colleagues as a source of information demonstrates that workplace interactions with fellow professionals contribute to the acquisition of cervical cancer knowledge.^51^ In contrast, the prevalence of media as a major information source across a substantial number of studies raises concern. This is because the information from media may lack rigour in its production, be unreliable to inform health knowledge, and may misrepresent current evidence on health issues.^61^ Again, despite the relevance of continuing medical education (CME) to meeting contemporary skills and information needs of health personnel, only a minute number of studies reported CME as an information source.^57^, ^59^ Such observation suggests possible low prioritisation of cervical cancer in the CME curriculum for health workers.

This study revealed that FHWs hold positive attitudes towards CCS. Research suggests that favourable attitudes towards CCS have a profound influence on CCS practice among individuals.^62^ Majority of the studies documented that a significant proportion of FHWs perceived screening as an important procedure, were willing to partake in screening, and intend to recommend screening to other eligible women.^53^, ^56^ While FHWs favourable attitude towards CCS is not unexpected due to their background, such attitudes play a significant role in creating a supportive environment that facilitates screening uptake among their colleagues and women in the general population.^34^ Furthermore, the observed disparity between willingness to recommend screening and actual recommendation practices among FHWs in Nigeria highlights the need to translate behavioural intentions into desired practices among this group.^50^

Regrettably, this study highlighted poor screening practices among FHWs despite possessing a high level of knowledge and good attitude towards CCS. This observation raises serious concerns as FHWs are expected to be champions of positive health behaviour and practices aimed at protecting and improving health.^53^ Even more perturbing was the observation of poor screening uptake among FHWs in facilities where services are readily available.^56^ Observed low uptake of CCS among a group perceived to be at the frontline of health protection portends abysmal outcomes for women in the general population who may lack appropriate knowledge. Additionally, such poor practice of CCS demonstrates that utilisation of screening is not entirely dependent on knowledge and attitudes but also influenced by broader factors.^49^ Moreover, it has been suggested that alongside knowledge and attitude, a complex interplay of socioeconomic and cultural factors that mediate consumption of health services could predict CCS uptake.^48^

A mix of health system and individual level barriers were identified as major reasons for not screening among FHWs. The preponderance of screening cost as a key barrier represented a key structural challenge impeding CCS uptake among FHWs. This finding agrees with previous evidence highlighting the significant impact of socioeconomic status on the uptake of screening.^63^, ^64^ The cost of CCS which ranges from $25 to 30 (₦10 250–₦12 300) could be prohibitive as payment for the service is mostly reliant on out‐of‐pocket spending.^30^, ^65^ Considering that the cost of CCS could be up to 41% of the monthly minimum wage (₦30 000), inability to afford screening by FHWs may result from poor remuneration, competing needs, and/or poor coverage of existing health insurance schemes.^50^ Financial constraints may also reflect wider economic issues in a country where more than 50% of the population lives below $2 daily.^66^ Other institutional challenges such as cumbersome nature of the procedure and lack of CCS recommendation highlight the need for revaluation of current practices to facilitate an environment that encourages screening uptake.

Predominant screening barriers at the individual level such as fear of positive result, low‐risk perception, lack of test awareness, and reluctance to screen are worrisome to be observed among FHWs. These findings are consistent with the results of systematic reviews conducted by Lim and Ojo^24^ and Black et al.^64^ Fear of positive result may emanate from either fatalistic beliefs that positive CCS result equals a death sentence or potential labelling due to perceived association of cervical cancer with promiscuity.^24^, ^67^ Equally concerning is observed low cervical cancer risk perception among FHWs who felt they were not susceptible to the disease. Such poor perception of the threat posed by cervical cancer may precipitate ignorance or reluctance to utilise CCS services as elicited in the majority of the reviewed studies. Consequently, cervical cancer may be detected among this group at advanced stages due to poor screening practices.^54^ Hence, it becomes pertinent that interventions must prioritise risk perception among this group to improve CCS.^68^

This study identified marital status, increasing age, awareness of screening methods, and physician recommendation as significant facilitators for CCS uptake among FHWs. These facilitators align with those documented in a similar systematic review by Black et al^64^ in Uganda. In some selected studies, younger and unmarried FHWs believe that only older and married women were at greater risk of developing cervical cancer.^46^, ^59^ In contrast, evidence suggests that older people may have better risk perception which subsequently facilitates the utilisation of preventive services such as screening.^52^ Again, being married may predispose women to a greater need for health services which include CCS. Furthermore, identifying physicians' awareness of screening methods and subsequent recommendation as facilitators of CCS uptake among FHWs highlights the need to leverage existing opportunities for cervical cancer education and screening recommendation.

### 4.1 Study limitations and strengths

It is advisable to keep several caveats in mind when interpreting the findings of this study due to inherent limitations. Although we searched for grey literature, the non‐inclusion of a few eligible primary studies due to their inaccessibility may weaken the overall conclusion of the study. Second, all studies included in this review were of quantitative cross‐sectional study designs. Cross‐sectional studies are susceptible to a spectrum of bias; exposure‐effect bias, recall bias, and response bias.^69^ Third, heterogeneity in outcome measurements across studies made comparison and summarisation of results difficult. Finally, the use of narrative synthesis which is largely dependent on the researcher interpretation of primary findings may introduce interpretive bias.

Nonetheless, this study possesses several strengths that improve the validity of drawn conclusions. First, the use of independent reviewers in study screening and selection, quality appraisal, and data extraction. Next, this study relied on a robust and exhaustive search strategy across selected databases and grey literature sources. Finally, we adhered to the UK Economic and Social Research Council's established principles guiding the conduct of narrative synthesis of data.

## 5 CONCLUSION

By identifying and synthesising results from available primary studies, this review provides robust evidence that can inform policy and programme initiatives directed at factors influencing CCS among FHWs in Nigeria. This study observed that a complex interplay of socioeconomic, structural, and individual factors influences CCS among FHWs in Nigeria. Equally important is the need to translate observed good knowledge and attitudes among this population into improved CCS. Hence, implementing holistic interventions targeting both the health system factors such as cost of screening and infrastructure, and individual factors such as low‐risk perception and fear of positive result affecting FHWs in Nigeria is critical to improving CCS outcomes. Consequently, improved screening practices among this group is likely to trigger a ripple effect of increased CCS utilisation among women who they come in contact with.

## CONFLICT OF INTEREST

The authors declare there is no conflict of interest.

## AUTHOR CONTRIBUTIONS

*Research Design, Search Strategy, Study Selection, Data Extraction, Quality Appraisal, Data Synthesis, Manuscript Writing, Manuscript Review and Editing*, O.E.; *Research Design, Quality Appraisal, Manuscript Review and Editing*, B.D.; *Research Design, Manuscript Review and Editing*, N.L.; *Research Design, Study Selection, Data Extraction, Data Synthesis, Manuscript Review and Editing*, A.S.; *Study Selection, Manuscript Review and Editing*, A.D.; *Research Design, Study Selection, Manuscript Review and Editing*, N.B.

## ETHICAL STATEMENT

No ethical approval was required as our study relied on the retrieval and analysis of already anonymised data from previous published studies.

## Data Availability

Data sharing is not applicable to this article as no new data were created or analyzed in this study.

## Appendix

**TABLE A1:** Sample search outcomes (Medline and CINAHL)

Search (S)	Search term/keywords	PIOS	Medline results	CINAHL results
S1	(MH “Allied Health Personnel+/OG”)	Population	2447	2829
S2	(MH “Health Personnel+”)	Population	510 397	579 215
S3	nurse*	Population	365 945	518 199
S4	female N5 (health OR healthcare OR health care) N5 (workers OR personnel OR professionals OR providers OR staff)	Population	1567	842
S5	(MH “Nurses+”)	Population	87 267	230 681
S6	S1 OR S2 OR S3 OR S4 OR S5	Population	738 739	875 909
S7	(MH “Uterine Cervical Neoplasms/DI/PC”)	Intervention	22 427	
S8	cervical screening	Intervention	12 685	5492
S9	pap* N5 (smear OR test*)	Intervention	28 311	6869
S10	“visual inspection” N5 (“acetic acid” OR “lugol* iodine”)	Intervention	629	210
S11	(MH “Colposcopy”)	Intervention	6274	1710
S12	Colposcopy	Intervention	9194	2345
S13	(MH “Early Detection of Cancer”)	Intervention	25 385	9121
S14	hpv testing	Intervention	3794	1150
S15	(vaginal OR cervical) N5 smear	Intervention	24 768	6861
S16	(vaginal OR pelvic) N5 exam*	Intervention	8754	2352
S17	cervical cytology	Intervention	5021	1105
S18	cytological screening	Intervention	757	77
S19	S7 OR S8 OR S9 OR S10 OR S11 OR S12 OR S13 OR S14 OR S15 OR S16 OR S17 OR S18	Intervention	94 572	24 900
S20	(MH “Health Knowledge Attitudes, Practice”)	Outcome	178 782	
S21	(MH “Health Knowledge Attitudes, Practice”)	Outcome	178 782	1925
S22	(MH “Attitude of Health Personnel+”)	Outcome	156 791	99 956
S23	(MH “Attitude to Health+”)	Outcome	414 103	158 294
S24	knowledge	Outcome	772 722	239 323
S25	(MH “Awareness”)	Outcome	20 009	
S26	Awareness	Outcome	157 048	72 065
S27	(MH “Perception+”)	Outcome	425 290	83 434
S28	Perception	Outcome	434 662	157 496
S29	attitudes	Outcome	420 370	334 654
S30	practices	Outcome	1 178 333	675 950
S31	enablers	Outcome	3245	2139
S32	determin*	Outcome	3 556 339	545 669
S33	predictors	Outcome	379 894	134 611
S34	difficult*	Outcome	629 147	152 346
S35	cost	Outcome	642 803	225 728
S36	(MH “Patient Acceptance of Health Care+”)	Outcome	150 887	
S37	(MH “Socioeconomic Factors+”)	Outcome	445 716	352 407
S38	access	Outcome	328 113	144 781
S39	socioeconomic	Outcome	216 875	107 592
S40	facilitators	Outcome	20 745	13 483
S41	promot*	Outcome	1 111 525	228 627
S42	uptake	Outcome	383 350	41 290
S43	challenges	Outcome	607 113	179 225
S44	obstacles	Outcome	48 460	12 364
S45	S20 OR S21 OR S22 OR S23 OR S24 OR S25 OR S26 OR S27 OR S28 OR S29 OR S30 OR S31 OR S32 OR S33 OR S34 OR S35 OR S36 OR S37 OR S38 OR S39 OR S40 OR S41 OR S42 OR S43 OR S44	Outcome	8 849 829	2 500 007
S46	Nigeria*	Setting	54 256	9287
S47	subsahara* africa	Setting	153	17
S48	sub sahara* africa	Setting	22 315	6472
S49	developing countr*	Setting	127 986	29 127
S50	low and middle income countr*	Setting	18 474	8728
S51	low resource setting*	Setting	4604	1821
S52	S46 OR S47 OR S48 OR S49 OR S50 OR S51	Setting	209 756	50 443
S53	(S6 AND S19 AND S45 AND S52)	PIOS	143	65

**TABLE A2:** Sample search outcome (Embase)

#	Query	Results
1	exp paramedical personnel/	525 389
2	exp health care personnel/	1 672 947
3	nurse*.mp. [mp = title, abstract, heading word, drug trade name, original title, device manufacturer, drug manufacturer, device trade name, keyword, floating subheading word, candidate term word]	436 419
4	(female adj5 (health or healthcare or health care) adj5 (workers or personnel or professionals or providers or staff)).mp. [mp = title, abstract, heading word, drug trade name, original title, device manufacturer, drug manufacturer, device trade name, keyword, floating subheading word, candidate term word]	41 319
5	exp nurse/	191 249
6	or/1–5	1 858 408
7	exp uterine cervix tumor/di, pc [Diagnosis, Prevention]	28 540
8	cervical screening.mp. [mp = title, abstract, heading word, drug trade name, original title, device manufacturer, drug manufacturer, device trade name, keyword, floating subheading word, candidate term word]	4281
9	(pap* adj5 [smear or test*]).mp. [mp = title, abstract, heading word, drug trade name, original title, device manufacturer, drug manufacturer, device trade name, keyword, floating subheading word, candidate term word]	40 172
10	(“visual inspection” adj5 [“acetic acid” or “lugol* iodine”]).mp. [mp = title, abstract, heading word, drug trade name, original title, device manufacturer, drug manufacturer, device trade name, keyword, floating subheading word, candidate term word]	1036
11	colposcopy/	12 538
12	Colposcopy.mp. [mp = title, abstract, heading word, drug trade name, original title, device manufacturer, drug manufacturer, device trade name, keyword, floating subheading word, candidate term word]	14 347
13	early cancer diagnosis/	8481
14	hpv testing.mp. [mp = title, abstract, heading word, drug trade name, original title, device manufacturer, drug manufacturer, device trade name, keyword, floating subheading word, candidate term word]	4219
15	([vaginal or cervical] adj5 smear).mp. [mp = title, abstract, heading word, drug trade name, original title, device manufacturer, drug manufacturer, device trade name, keyword, floating subheading word, candidate term word]	4586
16	([vaginal or pelvic] adj5 exam*).mp. [mp = title, abstract, heading word, drug trade name, original title, device manufacturer, drug manufacturer, device trade name, keyword, floating subheading word, candidate term word]	15 804
17	cervical cytology.mp. [mp = title, abstract, heading word, drug trade name, original title, device manufacturer, drug manufacturer, device trade name, keyword, floating subheading word, candidate term word]	5170
18	cytological screening.mp. [mp = title, abstract, heading word, drug trade name, original title, device manufacturer, drug manufacturer, device trade name, keyword, floating subheading word, candidate term word]	684
19	or/7–18	95 388
20	attitude to health/	117 813
21	exp health personnel attitude/	190 498
22	knowledge.mp. [mp = title, abstract, heading word, drug trade name, original title, device manufacturer, drug manufacturer, device trade name, keyword, floating subheading word, candidate term word]	987 762
23	awareness/	101 500
24	awareness.mp. [mp = title, abstract, heading word, drug trade name, original title, device manufacturer, drug manufacturer, device trade name, keyword, floating subheading word, candidate term word]	261 073
25	exp perception/	377 204
26	Perception.mp. [mp = title, abstract, heading word, drug trade name, original title, device manufacturer, drug manufacturer, device trade name, keyword, floating subheading word, candidate term word]	377 482
27	attitudes.mp. [mp = title, abstract, heading word, drug trade name, original title, device manufacturer, drug manufacturer, device trade name, keyword, floating subheading word, candidate term word]	154 176
28	practices.mp. [mp = title, abstract, heading word, drug trade name, original title, device manufacturer, drug manufacturer, device trade name, keyword, floating subheading word, candidate term word]	297 483
29	enablers.mp. [mp = title, abstract, heading word, drug trade name, original title, device manufacturer, drug manufacturer, device trade name, keyword, floating subheading word, candidate term word]	3936
30	determin*.mp. [mp = title, abstract, heading word, drug trade name, original title, device manufacturer, drug manufacturer, device trade name, keyword, floating subheading word, candidate term word]	4 951 537
31	predictors.mp. [mp = title, abstract, heading word, drug trade name, original title, device manufacturer, drug manufacturer, device trade name, keyword, floating subheading word, candidate term word]	388 708
32	difficult*.mp. [mp = title, abstract, heading word, drug trade name, original title, device manufacturer, drug manufacturer, device trade name, keyword, floating subheading word, candidate term word]	935 275
33	cost.mp. [mp = title, abstract, heading word, drug trade name, original title, device manufacturer, drug manufacturer, device trade name, keyword, floating subheading word, candidate term word]	944 491
34	exp patient advocacy/or patient attitude/	92 717
35	exp sociobiology/or socioeconomics/	161 632
36	access.mp. [mp = title, abstract, heading word, drug trade name, original title, device manufacturer, drug manufacturer, device trade name, keyword, floating subheading word, candidate term word]	548 036
37	socioeconomic.mp. [mp = title, abstract, heading word, drug trade name, original title, device manufacturer, drug manufacturer, device trade name, keyword, floating subheading word, candidate term word]	122 627
38	facilitators.mp. [mp = title, abstract, heading word, drug trade name, original title, device manufacturer, drug manufacturer, device trade name, keyword, floating subheading word, candidate term word]	23 299
39	promot*.mp. [mp = title, abstract, heading word, drug trade name, original title, device manufacturer, drug manufacturer, device trade name, keyword, floating subheading word, candidate term word]	1 477 929
40	uptake.mp. [mp = title, abstract, heading word, drug trade name, original title, device manufacturer, drug manufacturer, device trade name, keyword, floating subheading word, candidate term word]	539 149
41	challenges.mp. [mp = title, abstract, heading word, drug trade name, original title, device manufacturer, drug manufacturer, device trade name, keyword, floating subheading word, candidate term word]	396 902
42	obstacles.mp. [mp = title, abstract, heading word, drug trade name, original title, device manufacturer, drug manufacturer, device trade name, keyword, floating subheading word, candidate term word]	38 454
43	or/20–42	10 596 239
44	Nigeria*.mp. [mp = title, abstract, heading word, drug trade name, original title, device manufacturer, drug manufacturer, device trade name, keyword, floating subheading word, candidate term word]	50 728
45	subsahara* africa.mp. [mp = title, abstract, heading word, drug trade name, original title, device manufacturer, drug manufacturer, device trade name, keyword, floating subheading word, candidate term word]	295
46	developing countr*.mp. [mp = title, abstract, heading word, drug trade name, original title, device manufacturer, drug manufacturer, device trade name, keyword, floating subheading word, candidate term word]	142 878
47	(low and middle income countr*).mp. [mp = title, abstract, heading word, drug trade name, original title, device manufacturer, drug manufacturer, device trade name, keyword, floating subheading word, candidate term word]	27 268
48	low resource setting*.mp. [mp = title, abstract, heading word, drug trade name, original title, device manufacturer, drug manufacturer, device trade name, keyword, floating subheading word, candidate term word]	6447
49	or/44–48	216 996
50	6 and 19 and 43 and 49	2235

**TABLE A3:** Sample search outcome (SCOPUS)

#	Search terms	Results
14	((TITLE‐ABS‐KEY [“Health Personnel” OR nurse*]) OR (TITLE‐ABS‐KEY ((female) W/5 (health OR healthcare OR “health care”) W/5 (workers OR personnel OR professionals OR providers OR staff)))) AND ((TITLE‐ABS‐KEY [“Uterine Cervical Neoplasms” OR “cancer screening”]) OR (TITLE‐ABS‐KEY (pap* W/5 [smear OR test*])) OR (TITLE‐ABS‐KEY (“visual inspection” W/5 [“acetic acid” OR “lugol* iodine”])) OR (TITLE‐ABS‐KEY [colposcopy OR “early detection of cancer” OR “hpv testing”]) OR (TITLE‐ABS‐KEY ([vaginal OR cervical] W/5 smear)) OR (TITLE‐ABS‐KEY ([vaginal OR pelvic] W/5 exam*)) OR (TITLE‐ABS‐KEY [“cervical cytology” OR “cytological screening”])) AND (TITLE‐ABS‐KEY (knowledge OR attitudes OR awareness OR perception OR practices OR enablers OR determin* OR predictors OR diffcult* OR cost OR “patient acceptance” OR socioeconomic OR access OR facilitators OR promot* OR uptake OR challenges OR obstacles)) AND (TITLE‐ABS‐KEY (nigeria* OR “subsahara* africa” OR “sub sahara* africa” OR “developing countr*” OR “low and middle income countr*” OR “low resource setting”)) View Less	279
13	TITLE‐ABS‐KEY (nigeria* OR “subsahara* africa” OR “sub sahara* africa” OR “developing countr*” OR “low and middle income countr*” OR “low resource setting”)	447,121
12	TITLE‐ABS‐KEY (knowledge OR attitudes OR awareness OR perception OR practices OR enablers OR determin* OR predictors OR diffcult* OR cost OR “patient acceptance” OR socioeconomic OR access OR facilitators OR promot* OR uptake OR challenges OR obstacles)	21,457,461
11	(TITLE‐ABS‐KEY (“Uterine Cervical Neoplasms” OR “cancer screening”)) OR (TITLE‐ABS‐KEY (pap* W/5 (smear OR test*))) OR (TITLE‐ABS‐KEY (“visual inspection” W/5 (“acetic acid” OR “lugol* iodine”))) OR (TITLE‐ABS‐KEY (colposcopy OR “early detection of cancer” OR “hpv testing”)) OR (TITLE‐ABS‐KEY ((vaginal OR cervical) W/5 smear)) OR (TITLE‐ABS‐KEY ((vaginal OR pelvic) W/5 exam*)) OR (TITLE‐ABS‐KEY (“cervical cytology” OR “cytological screening”)) View Less	345,552
10	TITLE‐ABS‐KEY (“cervical cytology” OR “cytological screening”)	5,060
9	TITLE‐ABS‐KEY ((vaginal OR pelvic) W/5 exam*)	13,967
8	TITLE‐ABS‐KEY ((vaginal OR cervical) W/5 smear)	25,292
7	TITLE‐ABS‐KEY (colposcopy OR “early detection of cancer” OR “hpv testing”)	43,841
6	TITLE‐ABS‐KEY (“visual inspection” W/5 (“acetic acid” OR “lugol* iodine”))	800
5	TITLE‐ABS‐KEY (pap* W/5 (smear OR test*))	185,789
4	TITLE‐ABS‐KEY (“Uterine Cervical Neoplasms” OR “cancer screening”)	128,334
3	(TITLE‐ABS‐KEY (“Health Personnel” OR nurse*)) OR (TITLE‐ABS‐KEY ((female) W/5 (health OR healthcare OR “health care”) W/5 (workers OR personnel OR professionals OR providers OR staff)))	614,825
2	TITLE‐ABS‐KEY ((female) W/5 (health OR healthcare OR “health care”) W/5 (workers OR personnel OR professionals OR providers OR staff))	2,054
1	TITLE‐ABS‐KEY (“Health Personnel” OR nurse*)	613,409

**TABLE A4:** Sample search outcome (Web of Science)

#	Search history	Results
# 5	#4 AND #3 AND #2 AND #1 Indexes = SCI‐EXPANDED, SSCI, A&HCI, CPCI‐S, CPCI‐SSH, ESCI Timespan = All years	99
# 4	TOPIC: (nigeria* OR “subsahara* africa” OR “sub sahara* africa” OR “developing countr*” OR “low and middle income countr*” OR “low resource setting”) Indexes = SCI‐EXPANDED, SSCI, A&HCI, CPCI‐S, CPCI‐SSH, ESCI Timespan = All years	249,855
# 3	TOPIC: (knowledge OR awareness OR perception OR attitudes OR practices OR enablers OR determin* OR predictors OR difficult* OR cost OR “patient acceptance” OR socioeconomic OR access OR facilitators OR promot* OR uptake OR challenges OR obstacles) Indexes = SCI‐EXPANDED, SSCI, A&HCI, CPCI‐S, CPCI‐SSH, ESCI Timespan = All years	15,754,209
# 2	TOPIC: (“uterine cervical neoplasms” OR “cervical screening”) OR TOPIC: (pap* NEAR/5 (smear OR test*)) OR TOPIC: (“visual inspection” NEAR/5 (“acetic acid” OR “lugol* iodine”)) OR TOPIC: (Colposcopy OR “early detection of cancer” OR “hpv testing”) OR TOPIC: ((vaginal OR cervical) NEAR/5 smear) OR TOPIC: ((vaginal OR pelvic) NEAR/5 exam*) OR TOPIC: (“cervical cytology” OR “cytological screening”) Indexes = SCI‐EXPANDED, SSCI, A&HCI, CPCI‐S, CPCI‐SSH, ESCI Timespan = All years	131,073
# 1	TOPIC: (“health personnel” OR nurse*) OR TOPIC: (female NEAR/5 (health OR healthcare OR “health care”) NEAR/5 (workers OR personnel OR professionals OR providers OR staff)) Indexes = SCI‐EXPANDED, SSCI, A&HCI, CPCI‐S, CPCI‐SSH, ESCI Timespan = All years	226,839

**TABLE A5:** Excluded citations with reasons

S/n	Author‐date	Title	Reason
1	Nwobodo and Malami (2005)	‘Knowledge and practice of cervical screening among female health workers in Sokoto, North Western Nigeria’	Full text unavailable
2	Addah et al. (2012)	‘Knowledge, attitude and practice of cervical cancer screening – Papanicolaou test (Pap smear) among female health care providers in Port Harcourt’	Full text unavailable
3	Kabir et al. (2005)	‘Awareness and Practice of Cervical Cancer Screening among Female Health Professionals in Murtala Mohammed Specialist Hospital, Kano’	Full text unavailable
4	Aniebue and Aniebue (2010)	‘Awareness and practice of cervical cancer screening among female undergraduate students in a Nigerian university’	Different study population excluding FHWs
5	Ndikom and Ofi (2012)	‘Awareness, perception and factors affecting utilisation of cervical cancer screening services among women in Ibadan, Nigeria: a qualitative study’	Different study population excluding FHWs
6	Mbamara et al. (2011)	‘Knowledge, Attitude and Practice of Cervical Cancer Screening Among Women Attending Gynaecology Clinics in a Tertiary Level Medical Care Center in Southeastern Nigeria’	Different study population excluding FHWs
7	Okunowo and Smith‐Okonu (2020)	‘Cervical cancer screening among urban Women in Lagos, Nigeria: Focus on barriers and motivators for screening’	Different study population excluding FHWs
8	Ubajaka et al. (2015)	‘Knowledge of Cervical Cancer and Practice of Pap Smear Testing among Secondary School Teachers in Nnewi North Local Government Area of Anambra State, South Eastern Nigeria’	Different study population excluding FHWs
9	Obeidat et al. (2012)	‘Awareness, practice and attitude to cervical Papanicolaou smear among female health care workers in Jordan: Awareness to cervical Pap smear among health care workers’	Different study location – Not Nigeria
10	Gebreegziabher et al. (2016)	‘Factors Affecting the Practices of Cervical Cancer Screening among Female Nurses at Public Health Institutions in Mekelle Town, Northern Ethiopia, 2014: A Cross‐Sectional Study’	Different study location – Not Nigeria
11	Urasa and Darj (2011)	‘Knowledge of cervical cancer and screening practices of nurses at a regional hospital in Tanzania’	Different study location – Not Nigeria
12	Goyal et al. (2013)	‘Knowledge, attitude and practices about cervical cancer and screening among nursing staff in a teaching hospital’	Different study location – Not Nigeria
13	Silva de Brito et al. (2014)	‘Social support and cervical and breast cancer screening practices among nurses’	Different study location – Not Nigeria
14	Seyoum et al. (2017)	‘Utilisation of Cervical Cancer Screening and Associated Factors among Female Health Workers in Governmental Health Institution of Arba Minch Town and Zuria District, Gamo Gofa Zone, Arba Minch, Ethiopia, 2016’	Different study location – Not Nigeria
15	Turkistanlı et al. (2003)	‘Cervical Cancer Prevention and Early Detection ‐ The Role of Nurses and Midwives’	Different study location – Not Nigeria
16	Ekwunife and Lhachimi (2017)	‘Cost‐effectiveness of Human Papilloma Virus (HPV) vaccination in Nigeria: a decision analysis using pragmatic parameter estimates for cost and programme coverage’	Other intervention/focus on variables not relevant to this review.
17	Azuogu et al. (2019)	‘Appraisal of willingness to vaccinate daughters with human papilloma virus vaccine and cervical cancer screening uptake among mothers of adolescent students in Abakaliki, Nigeria’	Other intervention/focus on variables not relevant to this review.
18	Chigbu and Aniebue (2011)	‘The impact of community health educators on uptake of cervical and breast cancer prevention services in Nigeria’	Other intervention/focus on variables not relevant to this review.
19	Eze and Obiebi (2019)	‘Perspectives and practices of cancer screening among workers at a tertiary health facility in Nigeria: indications for adaptation and integration of best practices’	Other intervention/focus on variables not relevant to this review.
20	Akhigbe and Omuemu (2009)	‘Knowledge, attitudes and practice of breast cancer screening among female health workers in a Nigerian urban city’	Other intervention (Breast cancer screening)/focus on variables not relevant to this review.
21	Nyengidiki et al. (2019)	‘Does introduction of user fees affect the utilisation of cervical cancer screening services in Nigeria?’	Other intervention/focus on variables not relevant to this review.
22	Onyenwenyi and Mchunu (2019)	‘Primary health care workers' understanding and skills related to cervical cancer prevention in Sango PHC centre in south‐western Nigeria: a qualitative study’	Other intervention/focus on variables not relevant to this review.
23	Esan et al. (2019)	‘Awareness and utilisation of cervical cancer screening among women in an Urban area in Southwestern Nigeria’	Multiple Publication
24	Awoyesuku et al. (2019)	‘Determinants of cervical cancer screening via Pap smear among female staff in a tertiary hospital in Niger‐Delta of Nigeria’	Multiple publication
25	Dodo et al. (2016)	Exploring the Barriers to Breast and Cervical Cancer Screening in Nigeria: A Narrative Review	Review – only primary studies were considered for this paper.

**TABLE A6:** The AXIS quality assessment tool for cross‐sectional studies. Link to quality assessment outcome for included studies: Critical appraisal

	Question	Yes	No	Don't know/comment
*Introduction*				
1	Were the aims/objectives of the study clear?			
*Methods*				
2	Was the study design appropriate for the stated aim(s)?			
3	Was the sample size justified?			
4	Was the target/reference population clearly defined? (Is it clear who the research was about?)			
5	Was the sample frame taken from an appropriate population base so that it closely represented the target/reference population under investigation?			
6	Was the selection process likely to select subjects/participants that were representative of the target/reference population under investigation?			
7	Were measures undertaken to address and categorise non‐responders?			
8	Were the risk factor and outcome variables measured appropriate to the aims of the study?			
9	Were the risk factor and outcome variables measured correctly using instruments/measurements that had been trialled, piloted or published previously?			
10	Is it clear what was used to determined statistical significance and/or precision estimates? (e.g. p‐values, confidence intervals)			
11	Were the methods (including statistical methods) sufficiently described to enable them to be repeated?			
*Results*				
12	Were the basic data adequately described?			
13	Does the response rate raise concerns about non‐response bias?			
14	If appropriate, was information about non‐responders described?			
15	Were the results internally consistent?			
16	Were the results presented for all the analyses described in the methods?			
*Discussion*				
17	Were the authors' discussions and conclusions justified by the results?			
18	Were the limitations of the study discussed?			
*Other*				
19	Were there any funding sources or conflicts of interest that may affect the authors' interpretation of the results?			
20	Was ethical approval or consent of participants attained?			

**TABLE A7:** Data extraction tool. Link to data extraction document: Data extraction

S/n	Stage of the process	Information available
	Author(s), publication year	
	What was the hypothesis/question/aim of the study?	
	Study Design	
	Setting	
	Participants (sampling information)	
	Intervention or Issue	
	Main outcome measures related to cervical cancer screening E.g., Knowledge, attitude, practices, barriers, facilitators, etc.	

	Findings	
	Conclusion (main recommendations for practice or future research)	
	Strengths/limitations	
